# Effect of Norharmane *in vitro* on Juvenile Hormone Epoxide Hydrolase Activity in the Lower Termite, *Reticulitermes speratus*

**DOI:** 10.1673/031.008.1301

**Published:** 2008-02-21

**Authors:** Shuji Itakura, Satoshi Kawabata, Hiromi Tanaka, Akio Enoki

**Affiliations:** Department of Applied Biological Chemistry, Kinki University, Naka-Machi, Nara 631-8505, Japan

**Keywords:** caste differentiation, ergatoids, juvenile hormone metabolism, *Coptotermes formosanus*

## Abstract

The aromatic β-carboline norharmane was determined in workers, nymphs, and ergatoids of *Reticulitermes speratus* (Kolbe) (Isoptera: Rhinotermitidae) by gas chromatography/mass spectrometry. Norharmane levels in workers, nymphs, and ergatoids collected in May (∼4.3 ng/termite) were higher than those in November (∼0.2 ng/termite). Fluorescence of norharmane was observed in histological sections in whole animals and in the fat body. Norharmane, at a final concentration of 1 mM, stimulated juvenile hormone epoxide hydrolase (JHEH) activity of enzyme extract from ergatoids, but inhibited JHEH activity at higher concentrations. The elevated JHEH activity stimulated by norharmane should accelerate juvenile hormone metabolism in *R. speratus*.

## Introduction

Termite caste differentiation is a postembryonic developmental process. As in other species in the genus *Reticulitermes* (Hare 1934; Buchli 1958), the separation of two developmental lines after two larval instars (L1 and L2) has been observed in *Reticulitermes speratus* (Kolbe) (Isoptera: Rhinotermitidae) (Takematsu 1992). One pathway is the line of the worker caste, which comprises five instars (W1–W5), and the other pathway is the line of the nymphs, which comprises six instars (N1–N6) and is distinguished from other castes by the possession of wing buds. The last instar nymphs (N6) can molt into alates, and a pair of male and female alates establishes a new nest as primary reproductives. In due course, the primary reproductives are accompanied by, or replaced by, multiple neotenic reproductives (Thorne et al. 1999). In *R. speratus*, two types of neotenics emerge from each line of the workers and nymphs: ergatoids differentiate from workers (instar unidentified), and nymphoids differentiate from N3 to N6 nymphs (Kawamura 1982). Ergatoids are distinguished from workers by a narrower abdomen (Miyata et al. 2004), from senescent nymphs (N4–N5) by a lack of wing buds, and from last instar nymphs (N6) that have distinctly larger wing buds than those of NS (Takematsu 1992).

Caste differentiation in termites is strongly influenced by changes in juvenile hormone titer (Lüscher 1960). High titers of juvenile hormone lead to differentiation of immatures to the soldier caste, while low titers result in the development of neotenics (Yin and Gillot 1975). In *Reticulitermes flavipes*, exposure of worker termites to externally applied juvenile hormone III induces differentiation of presoldiers (Scharf et al. 2003).

Juvenile hormone III is methyl (2*E*,6*E*,10R)-10,11-epoxy-3,7,11-trimethyl-2,6-dodecadienoate. The primary routes of juvenile hormone metabolism in insects are ester hydrolysis to juvenile hormone acid and epoxide hydration to juvenile hormone diol. In the presence of both juvenile hormone esterase (JHE) and juvenile hormone epoxide hydrolase (JHEH), the primary metabolite juvenile hormone acid or juvenile hormone diol is metabolized to juvenile hormone diol-acid (Share and Roe 1988).

Norharmane, which is located in the hemolymph (Siderhurst et al. 2005a), has antimicrobial activity against the entomopathogenic fungus *Metarhizium anisopliae* (Siderhurst et al. 2005b). Norharmane also is a fluorescent chromophore and has autophototoxic activity in *R. flavipes, R. tibialis*, and *R. virginicus* (Siderhurst et al. 2005c, 2006).

Microsomal epoxide hydrolase activity is stimulated by norharmane (Bulleid and Craft 1984). As stated above, one of the primary routes of juvenile hormone metabolism is epoxide hydration by JHEH. Lepidopteran JHEH is expressed in the fat body and the gut (Gilbert et al. 2000). Produced in the corpora allata, juvenile hormone is transported to target cells via insect hemolymph (Gilbert et al. 2000). These relationships between norharmane, JHEH, and juvenile hormone raise an untested hypothesis that norharmane in the hemolymph could stimulate JHEH activity in *Reticulitermes* termites, and that an elevated JHEH activity could cause a decrease in juvenile hormone titer, which may affect caste differentiation. This simple explanation is supported by the development of alates in response to decreasing amounts of juvenile hormone (Henderson 1998). It also appears to be supported by evidence that nymphal formation occurs when juvenile hormone levels are low in *Coptotermes formosanus* (Isoptera: Rhinotermitidae) (Henderson 1998). In this species, nymphs (N1–N5) can be formed from any of the worker stages (W1–W5) and subsequently give rise to alates or brachypteroid neotenics (Raina et al. 2004). In this study, we have attempted to obtain evidence that norharmane is involved in juvenile hormone metabolism in *R. speratus*. This has been done by localization and quantitative analysis of norharmane in *R. speratus*, and by assessing the effect of norharmane on the activity of JHEH and JHE.

## Materials and Methods

### Insects

*R. speratus* individuals were collected from a wild colony located in an infested wood in the Wakayama Prefecture, Japan, in April 2006 and 2007 and were maintained in the laboratory at 26°C with their nest materials and with blocks of *Pinus densiflora* Siebold and Zucc (Pinals: Pinaceae) as the food source. Non-reproductives, nymphs with wing buds on the thorax, apterous workers, neotenic reproductives, and nymphoids differentiated from nymphs and ergatoids from workers as described by Miyata et al. (2004), were collected from the colony maintained in the laboratory in May and November 2007. Larvae, nymphs, soldiers, workers, ergatoids, and nymphoids were also collected from the colony maintained in the laboratory in June and July 2007. *C. formosanus* individuals, workers and nymphs, were collected from a nest that had been maintained with blocks of *P. densiflora* at 26°C in our laboratory for 6 years.

### Chemicals

**Figure 1.**
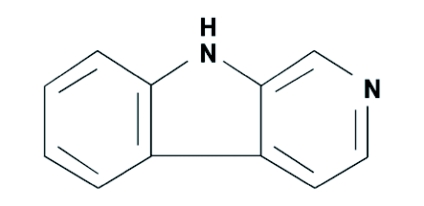
Norharmane: 9H-pyrido[3,4-b]indole.

Norharmane (Figure 1) was purchased from Sigma-Aldrich (www.sigmaaldrich.com). Labeled juvenile hormone III (647.5 GBq/mmol, 3H at C-10) was obtained from Perkin Elmer Life Science (www.perkinelmer.com). The radiochemical purity was >99%. Unlabeled juvenile hormone III (Sigma-Aldrich) was mixed with the labeled juvenile hormone III to give a final substrate concentration of 0.5 mM in ethanol having 8000 cpm/µl. 3-Octylthio-1,1,1-trifluoro-2-propanone (OTFP) was synthesized by the addition of equal molar quantities of *n*-octyl mercaptan to 3-bromo-1,1,1-trifluoropropanone (Wako Pure Chemical Industries, www.wako-chem.co.jp) in carbon tetrachloride as previously described (Hammock et al. 1984). After vacuum distillation, OTFP was dissolved in ethanol to give a final concentration of 10 mM. The mass spectrum of OTFP, *m*/z (relative intensity 96) 256 M^+^ (1), 199 (1), 171 (0.5), 159 (22), 145 (60), 129 (4), 69 (100), was consistent with the results reported by Hammock et al. 1984.

### Extraction for chemical analysis

One thousand individuals (∼2.6 g) of *R. speratus*, collected in May, consisting of workers, nymphs, and ergatoids, were ground in a mortar and extracted in methanol. One thousand workers (∼2.1 g) and 500 ergatoids (∼1.6 g) of *R. speratus* collected in November were separately homogenized and extracted in methanol. The methanol extract was filtered and the solvent was removed under decompression to give a sticky yellow residue that fluoresced under UV light. The residue was subjected to thin layer chromatography and gas chromatographic/mass spectrometric (GC/MS) analyses. The UV source for observations of termite fluorescence was a B104 (6V, 4W) lamp (Sato Shouji, www.ureruzo.com) equipped with a NVF4T5BLB black light bulb (output 300–400 nm). For *C. formosanus*, 1000 workers (∼3.3 g) and 500 nymphs (∼3.7 g) were separately ground and extracted in methanol, then subjected to GC/MS analyses.

### Preparation of enzyme extract

Twelve workers and ergatoids of *R. speratus* collected in November 2006 and in June and July 2007 were homogenized, respectively, in 1.2 ml of sodium phosphate buffer (pH 7.4, 200 mM), containing 0.01% phenylthiourea, and 10% sucrose (Share and Roe 1988). All operations were performed at 0–4°C.

### Microscopic observations

Fluorescence in individuals of *R. speratus* and *C. formosanus* was observed using a SZ260 stereoscopic zoom microscope (Olympus, Tokyo, Japan) under UV light. The images were captured using a Fine Pix 600Z digital camera (Fujifilm, www.fujifilm.com). For histological observations, individuals of *R. speratus* were embedded into a Tissue-Tek O.C.T. 4583 compound (Sakura Finetechnical, www.sakura-finetek.com) in a refrigerant of hexane and solid carbon dioxide (dry ice). Serial sections (10–15 µm) were processed using a CM 1850 cryostat (Leica Microsystems, www.leica-microsystems.com) and stained with hematoxylin and Sudan III. As norharmane dissolves readily in organic solvents, the Sudan III stain, which was dissolved in 70% ethanol, was used with caution. To avoid extraction of norharmane from tissues on slides, the tissues were stained with Sudan III for a few minutes, followed by hematoxylin, or the tissues were stained with Sudan III for 10 min and washed briefly with 50% ethanol. Tissues on slides were observed using an Eclipse 80i microscope (Nikon, www.nikon.com) under white light plus external UV light from the UV source. The images were captured using a DS-5MC CCD camera with a DS-U1 controller and an ACT-2U program (Nikon).

### Thin layer chromatographic and GC/MS analyses of norharmane

Thin layer chromatography was performed on precoated Silica Gel 60 TLC plates (Merck, www.merck.com). Thin layer plates were developed using a 75:15:10 n-butanol/water/formic acid solvent system (Stachel et al. 1999) and visualized either by fluorescence under UV light or by spraying with a 13% sulfuric acid and 2% copper (II) sulfate solution followed by heating. GC/MS analysis was performed on a JEOL (www.jeol.com) JMS-K9 mass detector interfaced with an Agilent Technology 6890N Network GC system equipped with a Phenomenex Zebron ZB/5ms column (30 m, 0.25 mm i.d., 0.25 µm film thickness). The temperature program was ramped from 160°C to 220°C at 5°C/min with a 2 min start delay. One µl of the methanol extract of the termites and 20 ppm norharmane as an external standard were injected using an Agilent (www.agilent.com) 7683 automatic liquid sampler. Quantitative analysis was performed by quantitative selected ion monitoring (SIM) at m/z 168 and a single point calibration curve method using the Quant Program in MS-66010 MPR software (JEOL). Gas chromatographic/flame ionization detector (GC/FID) analysis performed on a Shimadzu GC-2010 gas chromatograph failed to detect 20 ppm norharmane, though 2000 ppm norharmane was successfully detected. Sensitive GC/MS was, therefore, adopted for quantitative analysis. GC/MS analysis was separately conducted in May and November. Quantitative analysis of an external standard of 20 ppm norharmane in May and November had a margin of error of ∼5%.

### Assay of JHEH and JHE

The activity of JHEH and JHE in extracts from workers and ergatoids of *R. speratus* was determined by a partition assay following juvenile hormone acid, juvenile hormone diol, and juvenile hormone diol-acid production from ^3^H-labeled juvenile hormone III substrate (Share and Roe 1988). Each enzyme extract (100 µl) was pre-incubated with 1 µl of ethanol for the determination of total metabolism of JHEH and JHE, or 1 µl of 10 mM OTFP for JHEH activity, for 10 min at 30°C. Reactions were initiated by the addition of 1 µl of 3H-labeled juvenile hormone III substrate (0.5 mM, 8000 cpm). After incubation for 15 min at 30°C, methanol (300 µl) and iso-octane (250 µl) were added to the samples in the ice bath. Each sample was vigorously vortexed, followed by centrifugation at 1000 *g* for 5 min. Aliquots of 100 µl from the top and bottom phases were recovered and the radioactivity of each fraction was analyzed using a liquid scintillation counter (LSC-5100, Aloka, Tokyo, www2.aloka.co.jp). The volumes of iso-octane (top phase) and aqueous methanol (bottom phase) were 250 µl and 400 µl, respectively, and the percentage juvenile hormone III in the aqueous methanol phase was 26.5 (*n* = 5). The percentage metabolism was calculated using the following formula:

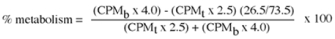

Juvenile hormone metabolism was calculated from the product of the decimal percentage metabolism, the moles of juvenile hormone III per assay (0.5 nmol) and incubation time. One unit of JHEH and JHE activity is defined as the amount of enzyme that metabolizes 1 µmol of juvenile hormone into juvenile hormone diol, juvenile hormone acid, and juvenile hormone diol-acid per minute.

### Thin layer chromatographic analysis of juvenile hormone III metabolites

Juvenile hormone III diol was produced by incubation of juvenile hormone III (a final concentration of 0.8 mM) in 0.5 M sodium acetate buffer (pH 4.0) containing 25 µg/ml of 2,6-di-*t*-butyl-4-methylphenol and 0.5% ethanol in darkness at 40°C for 24 h (Mumby and Hammock 1979). Juvenile hormone III acid was produced by incubating juvenile hormone III in methanol: 1 N NaOH (1:1, v/v) in darkness at 30°C for 18 h (Li et al. 2003). Juvenile hormone diol and juvenile hormone acid standards were stored as ethanol solutions at -20°C until used. Each enzyme extract (100 µl) was pre-incubated for 10 min at 30°C with 1 µl of ethanol for the determination of juvenile hormone III metabolites, or 1 µl of 5 mM or 10 mM OTFP for the assessment of efficacy of OTFP to inhibit JHE and JHEH activities. Reactions and partitions using methanol and iso-octane were conducted as described above, except that 3H-labeled juvenile hormone III (0.5 mM, 8000 cpm) was replaced by 3H-labeled juvenile hormone III (0.5 mM, 160000 cpm). After centrifugation, a 100 µl sample was taken from the bottom phase, and a 10 µl sample (1 µl 10 times) was directly spotted on the thin layer plate (Silica Gel 60, Merck). The plate was developed in hexane/EtOAc/AcOH (66:33:2) (Touhara and Prestwich 1993), and analyzed by radio-thin layer scanning using an Image Analyzing System (FLA-7000, Fujifilm) and Imaging plate (BAS-TR2040, Fujifilm). No radioactive spot was detected by the image analyzing system, though the imaging plate was exposed to the thin layer plate for 4 h, 18 h, and 48 h. The silica gel with 3H-labeled juvenile hormone III metabolites was removed from the thin layer plate at the *Rf* values for juvenile hormone III diol (*Rf* = 0.40) and juvenile hormone III acid (*Rf* = 0.72), and the radioactivity of each spot was analyzed by a liquid scintillation counter. The *Rf* values were determined on the same thin layer plate, onto which cold juvenile hormone III diol and juvenile hormone III acid standards had also been spotted and developed. They were visualized by a sulfuric acid and copper (II) sulfate solution as mentioned above.

**Figure 2.**
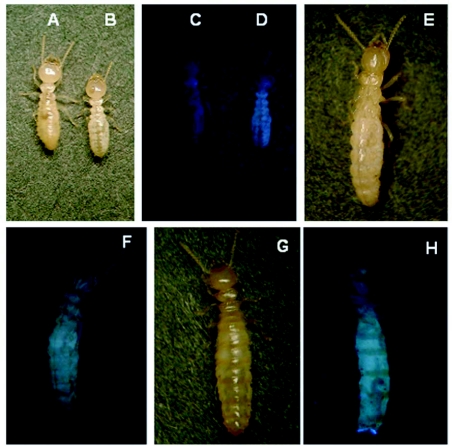
Fluorescence in *R. speratus*. Worker (A, C) of *Coptotermes formosanus* as a control and worker (B, D), nymph (E, F), and ergatoid (G, H) of *Reticulitermes speratus* under white light (A, B, E, G) and UV (output 300–400 nm) light (C, D, F, H).

### Effect of norharmane on juvenile hormone III metabolism

The determination of total metabolism and JHEH activity was carried out as described above, except that 1 µl of 10, 100, 300, and 500 mM norharmane in ethanol, or 1 µl of ethanol (control), was added to each enzyme extract before pre-incubation. Final concentrations of 0.1, 1, 3, and 5 mM norharmane were determined on the basis of the fact that 1 mM norharmane stimulated microsomal epoxide hydrolase activity (Bulleid and Craft 1984).

**Figure 3.**
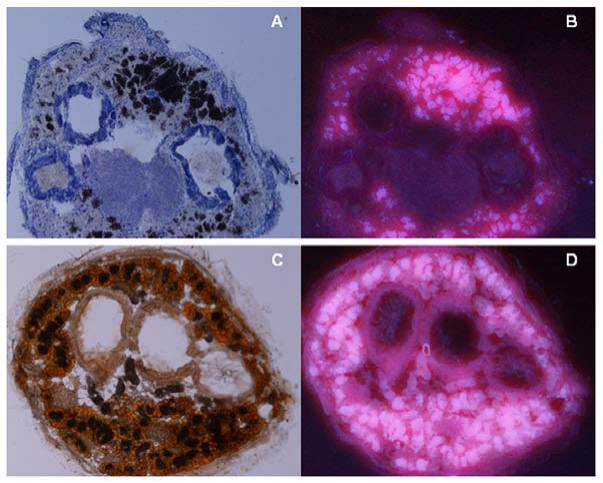
Fluorescence in abdominal cross sections of (B) worker and (D) ergatoid of *Reticulitermes speratus* under UV light. Abdominal cross sections of (A) worker stained with Sudan III and hematoxylin and (C) ergatoid stained with Sudan III under white light. Fat appeared orange by Sudan III stain.

**Figure 4.**
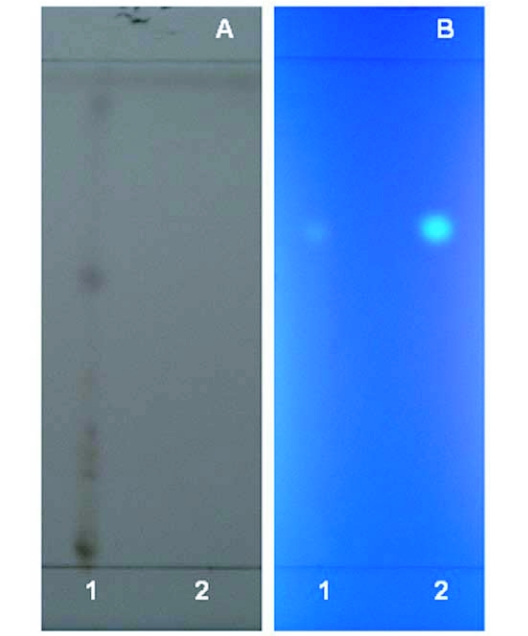
Thin layer chromatographic analysis of UV chromophore from *Reticulitermes speratus*. Spots on thin layer chromatographic plates were visualized by (A) spraying with a 13% sulfuric acid and 2% copper (II) sulfate solution followed by heating, and (B) fluorescence under UV light. Lane 1: methanol extract from workers and ergatoids of *R. speratus* collected in May. Lane 2: authentic norharmane. Note only one fluorescent spot (*R_f_* = 0.68) in methanol extract from *R. speratus*.

**Figure 5.**
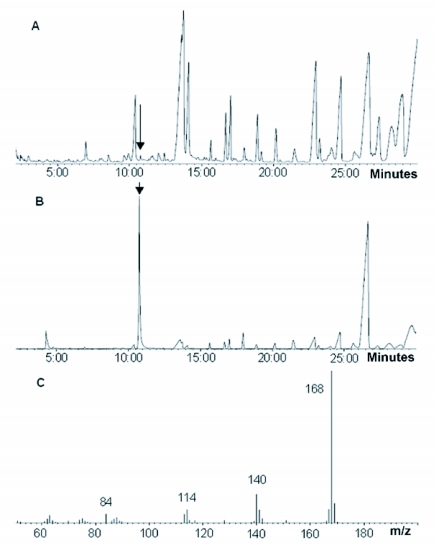
GC/MS analysis of methanol extract from workers and ergatoids of *Reticulitermes speratus*. (A) Total ion chromatograph, (B) Extracted ion chromatograph (*m*/*z* 168), (C) Mass spectrum of the arrowed peak (rt = 10.46 min).

## Results

### Localization of norharmane

Observations of workers, nymphs, ergatoids, and nymphoids of *R. speratus* using the stereoscopic zoom microscope showed blue fluorescence under UV light. The most intense fluorescence was observed in the abdomen of nymphs and ergatoids, as well as in the thorax and abdomen of workers (Figure 2). Approximately half of the non-reproductives, which consist of workers and nymphs, of *R. speratus* exhibited strong fluorescence similar to that of the worker shown (Figure 2-D) under UV light. In contrast, little fluorescence under UV light was observed in workers of *C. formosanus*, a member of the family Rhinotermitidae to which *R. speratus* also belongs (Figure 2-C). Histological observations under white and UV light showed that fluorescence was observed in fat bodies that had been stained an orange color with Sudan III (Figure 3). Fluorescence was not observed in the gut and cuticle of workers and ergatoids.

### Thin layer chromatographic and GC/MS analyses of norharmane

Thin layer chromatographic analyses of the methanol extract from a blend of workers, nymphs, and ergatoids of *R. speratus* collected in May showed a single bright spot, *Rf* = 0.68, that was consistent with the *Rf* of authentic norharmane visualized under UV light (Figure 4). As shown in Figure 5, GC/MS of the compound from the methanol extract of *R. speratus* at rt = 10.46 min showed peaks at *m*/*z* 168 M^+^ (100), 140 (19), 114 (9), and 84 (7), matching synthetic norharmane (rt = 10.37). The GC/MS quantitative analysis showed that individuals of *R. speratus* collected in May contained approximately 4.3 ng of norharmane per termite, but the norharmane level decreased to approximately a twentieth of that value in November (Table 1). The norharmane level of *R. speratus* workers was similar to that of ergatoids in November (Table 1). No norharmane was detected in workers of *C. formosanus*, but a trace of norharmane was detected in nymphs of *C. formosanus* (0.01 ng/termite).

**Table 1.**
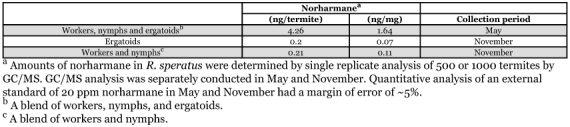
Norharmane levels in *Reticulitermes speratus*.

### JHEH and JHE

**Figure 6.**
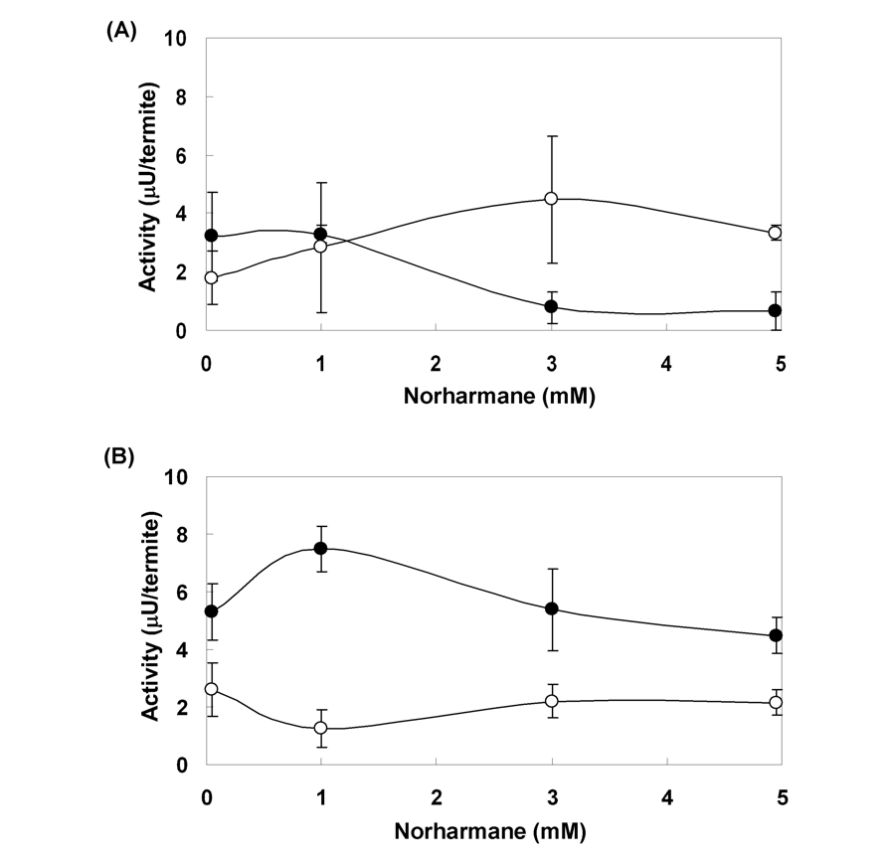
Relationship between JHEH or JHE activity and concentration of norharmane in (A) workers and (B) ergatoids of *Reticulitermes speratus*. Open circle: JHE activity. Closed circle: JHEH activity. Error bars represent 95% confidence intervals. Each measurement was replicated three times.

Addition of the selective JHE inhibitor OTFP to termite homogenates enabled assay of JHEH. Thin layer analysis of 3H-labeled juvenile hormone metabolites using a liquid scintillation counter showed that metabolites of juvenile hormone III by termite homogenates were juvenile hormone III diol and juvenile hormone III acid (data not shown) and that a final concentration of 0.1 mM OTFP depressed JHE activity of termite homogenate to ∼10% (the efficacy of inhibition was 89.0 ± 2.8%, n = 3) and led to depression of JHEH activity by 23.0 ± 3.9% (n = 3). Thus, JHEH activity, as determined by the partition assay used in the present study, contained modest JHE activity. The primary routes of juvenile hormone metabolism in homogenates of *R. speratus* are epoxide hydration and ester hydrolysis. Thus, the difference between total metabolism (ethanol-treated homogenate) and epoxide hydrolase activity (OTFP-treated homogenate) represented ester hydrolysis. The activity of JHEH was greater than JHE activity: 3.21 ± 0.48 µU/termite in the worker homogenate and 5.29 ± 0.31 µU/termite in the ergatoid homogenate (Table 2). JHEH activity varied among castes of termite. JHEH activity of neotenics as ergatoids and nymphoids was greater than that of other castes as larvae, nymphs, soldiers, and workers (Table 2). Administration of 1 mM norharmane significantly promoted JHEH activity in ergatoid homogenate (Table 2). However, inhibition of JHEH activity was observed at higher concentrations of norharmane (Figure 6). The difference in JHEH activity between worker homogenates to which 0 mM or 1 mM norharmane was added was not significant, nor was the difference in nymph and soldier homogenates (Table 2).

**Table 2.**
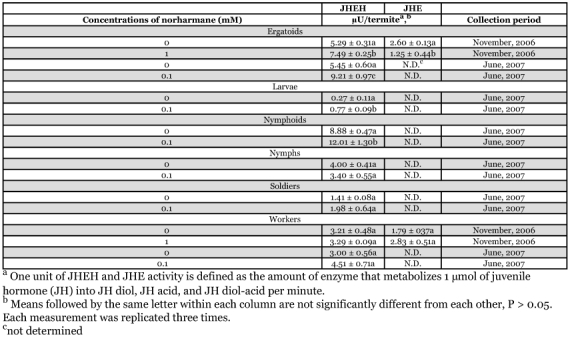
JH epoxide hydrolase (JHEH) and JH esterase (JHE) activity of *Reticulitermes speratus* ± SEM.

## Discussion

A single fluorescent chromophore was observed in *R. speratus* by thin layer analysis. A spot of moderate intensity at the origin and several weakly fluorescent spots on the thin layer plate, which have been reported in *R. flavipes, R. tibialis*, and *R. virginicus* (Siderhurst et al. 2005c), were not observed in *R. speratus*. The fluorescent chromophore was identified as norharmane in *R. speratus* and has also been identified in other *Reticulitermes* termites (Siderhurst et al. 2005c) by GC/MS analysis. The norharmane level in workers, nymphs, and ergatoids of *R. speratus* collected in May (∼4.3 ng/termite) was higher than that of workers of *R. flavipes* (1.3 ng/termite), *R. tibialis* (1.2 ng/termite), and *R. virginicus* (1.1 ng/termite) (Siderhurst et al. 2005c), but unexpectedly decreased in November (to ∼0.2 ng/termite). A rising level of norharmane was observed in May, when colony expansion by swarming occurs in *R. speratus*. This correlation raises the question of whether norharmane is involved in the metamorphoses of *R. speratus*, as discussed below.

Histological observations in this study showed that norharmane is mainly in the fat body of *R. speratus*. The biosynthetic origin of norharmane in *Reticulitermes* termites was reported to be the endosymbionts (Siderhurst et al. 2005a). Although the mechanism for transportation of norharmane from gut fluid, where endosymbionts are harbored, into the hemolymph of termites is not known, norharmane transported into the hemolymph should be carried into lipophilic organs such as the fat body, because lipophilic norharmane is almost insoluble in the aqueous hemolymph of termites. Our results do not contradict the observation that norharmane is contained in the internal fluids of *Reticulitermes* termites (Siderhurst et al. 2005a).

Although it is not clear whether JHEH is produced in the fat body of termites, the JHEH of the cabbage looper, *Trichoplusia ni*, is expressed in the fat body (Haris et al. 1999). As mentioned above, norharmane is condensed in the fat body of *R. speratus*, where a gene encoding JHEH could be expressed. This finding was investigated by assaying the effect of norharmane on the JHEH activity of enzyme extracts from *R. speratus* workers and ergatoids.

JHEH activity was significantly stimulated by norharmane when 0.1 or 1 mM norharmane was added to an extract of enzymes from *R. speratus* ergatoids and nymphoids. The ergatoids and nymphoids also had higher initial JHEH activity than the initial activity of enzyme extracts from larvae, nymphs, soldiers, and workers. It appears that the stimulatory effect of norharmane on JHEH activity occurs only at limited concentrations of norharmane. As shown in Table 1, an individual of *R. speratus* contained 1.64 ng of norharmane per mg-termite in May, indicating that ∼0.01 mM norharmane could be present in the termite (density of the termite was regarded as 1 g/ml). This low level of norharmane in *R. speratus* could not inhibit JHEH activity, but could stimulate JHEH activity in the termite. To the best of our knowledge, this is the first report that norharmane stimulates JHEH activity in insects.

JHEH from fat tissue of *T. ni* is a microsomal epoxide hydrolase belonging to the α,β-hydrolase enzyme family (Haris et al. 1999). The structural similarity among family member is very high (Ollis et al. 1992; Haris et al. 1999). Although the JHEH of *R. speratus* has not been characterized, if it is an epoxide hydrolase of the α,β-hydrolase enzyme family, the stimulatory effect of norharmane on JHEH observed in this study is consistent with the stimulation of microsomal epoxide hydrolase activity by norharmane reported by Bulleid and Craft (1984).

As stated earlier, the development of aletes is promoted in response to a decrease in juvenile hormone titer in lower termites, and it has been hypothesized that nymphal formation in *C. formosanus* occurs when juvenile hormone levels are low (Henderson 1998). Elevated JHEH activity causes a decrease in the juvenile hormone titer of *R. speratus*, but it is not yet possible to explain the function of norharmane in caste differentiation of *R. speratus*.

## Abbreviations

JHEH -: juvenile hormone epoxide hydrolase
JHE -: juvenile hormone esterase
OTFP -: 3-octylthio-1,1,1-trifluoro-2-propanoney
GC/MS -: gas chromatography/mass spectrometry
